# Establishment of ultrasound-responsive SonoBacteriaBot for targeted drug delivery and controlled release

**DOI:** 10.3389/fbioe.2023.1144963

**Published:** 2023-02-24

**Authors:** Meng Du, Ting Wang, Renjie Feng, Penghui Zeng, Zhiyi Chen

**Affiliations:** ^1^ The First Affiliated Hospital, Medical Imaging Centre, Hengyang Medical School, University of South China, Hengyang, Hunan, China; ^2^ Institute of Medical Imaging, Hengyang Medical School, University of South China, Hengyang, China; ^3^ The Seventh Affiliated Hospital, Hunan Veterans Administration Hospital, Hengyang Medical School, University of South China, Changsha, Hunan, China

**Keywords:** ultrasound, drug delivery, controlled release, biohybrid microbots, bacteria

## Abstract

Bacteria-driven biohybrid microbots have shown great potential in ca^n^cer treatment. However, how precisely controlling drug release at the tumor site is still an issue. To overcome the limitation of this system, we proposed the ultrasound-responsive SonoBacteriaBot (DOX-PFP-PLGA@*EcM*). Doxorubicin (DOX) and perfluoro-n-pentane (PFP) were encapsulated in polylactic acid-glycolic acid (PLGA) to form ultrasound-responsive DOX-PFP-PLGA nanodroplets. Then, DOX-PFP-PLGA@*EcM* is created by DOX-PFP-PLGA amide-bonded to the surface of *E. coli MG1655* (*EcM*). The DOX-PFP-PLGA@*EcM* was proved to have the characteristics of high tumor-targeting efficiency, controlled drug release capability, and ultrasound imaging. Based on the acoustic phase change function of nanodroplets, DOX-PFP-PLGA@*EcM* enhance the signal of US imaging after ultrasound irradiation. Meanwhile, the DOX loaded into DOX-PFP-PLGA@*EcM* can be released. After being intravenously injected, DOX-PFP-PLGA@*EcM* can efficiently accumulate in tumors without causing harm to critical organs. In conclusion, the SonoBacteriaBot has significant benefits in real-time monitoring and controlled drug release, which has significant potential applications for therapeutic drug delivery in clinical settings.

## Introduction

Over the decades, nanotechnology has been heavily researched for anti-cancer drug delivery applications (Yang and Gao, 2017). However, the heterogeneity of tumor vascular distribution and the passive targeting of drug-loaded nanoparticles lead to a restricted drug aggregation effect in the tumor area (Park et al., 2019; van der Meel et al., 2019). While bacteria-driven biohybrid microbots as tumor-targeting drug delivery systems can overcome the above barrier, have shown great potential in cancer treatment (Sonntag et al., 2019; Chen et al., 2020; Moreno and Baeza, 2022). In contrast to passive transport, bacteria use their flagella to migrate for longer periods in the tumor area, which makes bacteria promising carrier candidates for anti-cancer drug delivery (Gujrati et al., 2014; Hosseinidoust et al., 2016; Raj and Das, 2016; Chen et al., 2019). Although bacteria-driven biohybrid microbots are beneficial to carry drugs to tumor localization, there is still a pressing need to find a solution for precisely controlling drug release in the tumor site (Guo et al., 2020).

Control over the drug vehicles while tracking their localization is essential to increase the drug delivery efficiency in cancer treatment, as it helps to promote the precision of controlled drug release significantly (Guo et al., 2020; Zhang et al., 2021). To date, several stimulus models have been studied for controlled drug release, including endogenous stimuli [i.e., pH (Wu et al., 2019), enzyme, glucose, and glutathione (Shi et al., 2019)] and exogenous stimuli [X-rays (Fan et al., 2019; Li et al., 2020; Yang et al., 2022), light (Jin et al., 2019), and ultrasound (Roovers et al., 2019; Entzian and Aigner, 2021)]. In previous studies, the biological differences between the tumor microenvironment and normal regions were used to design the endogenous stimuli controllable drug release models. For instance, *E. coli* was used in earlier experiments along with magnetic particles that carried the chemotherapy drug doxorubicin. These multifunctional bacteria-driven microbots exhibited a desirable pH-responsive drug release for targeted tumor treatment (Park et al., 2017; Xie et al., 2017; Xie et al., 2018). However, due to the complexity and heterogeneity of the physiological environment in tumors, the regulation and release of drugs through an endogenous stimulus are unstable (Erkoc et al., 2019).

Compared with the above endogenous stimuli, local exogenous stimuli has the advantages of subjective controllability and less toxicity to the body. Therefore, some methods for drug delivery, which could be triggered under exogenous physical stimuli, such as X-rays (Fan et al., 2019; Li et al., 2020), light (Jin et al., 2019), and ultrasound (Roovers et al., 2019; Entzian and Aigner, 2021), have been investigated. For instance, Sentürk et al. (2020) employed red and far-red lasers to discharge cargo from bacteria-driven microbots selectively, but the finite depth of light penetration into deep tumor tissue might impede its further application. Compared to it, ultrasound has the advantage of high penetration, high safety, and relatively high spatiotemporal resolution (Huang, 2020). When ultrasound is combined with carriers (i.e., PFP) that respond to ultrasound, it is possible to perform ultrasound imaging (Long et al., 2021), as well as achieve non-invasive carrier-controlled drug release for precision responsiveness. It is proven that ultrasound has been used to track drugs throughout the body as well as to cause their release from drug carriers (De Cock et al., 2015). Currently, it has been used extensively in neural regulation (Ye et al., 2018) and ultrasound-targeted microbubble destruction (UTMD) (Zhu et al., 2018).

**FIGURE 1:**
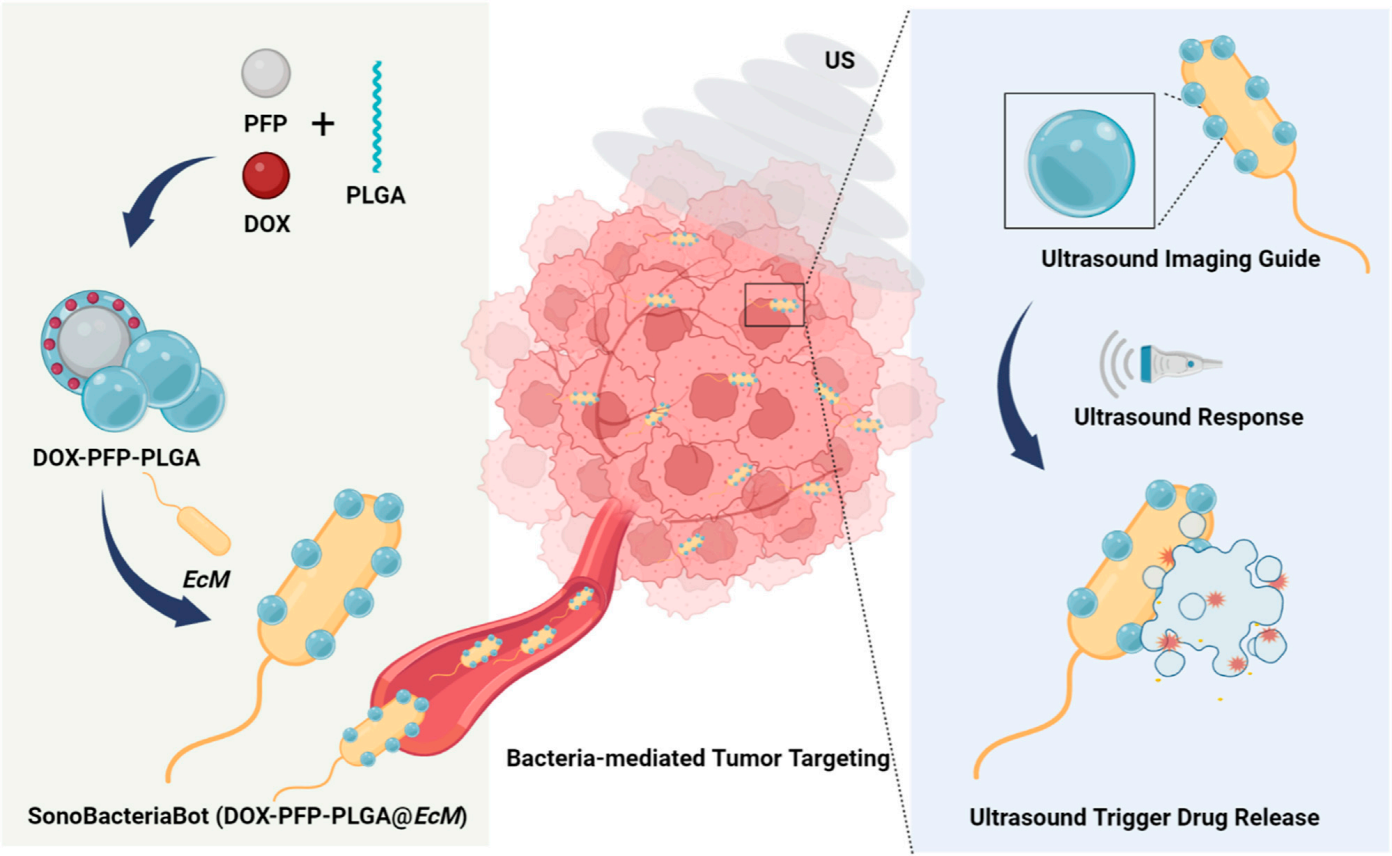
Schematic illustration of an imaging-guided US-responsive drug delivery system based on SonoBacteriaBot (DOX-PFP- PLGA@*EcM*) (Created with Biorender.com).

Herein, as shown in Figure 1, we describe the ultrasound-responsive SonoBacteriaBot, a new bacteria-driven drug delivery system for ultrasound-regulated drug release and imaging guidance. Using perfluoro-n-pentane (PFP) as the core, phase-change drug-loaded nanoparticles were prepared and attached to bacteria to construct the SonoBacteriaBot. In this system, the chemotherapy drug doxorubicin (DOX) is introduced for a drug release effect study. Encapsulating PFP and DOX in polylactic acid-glycolic acid (PLGA) to form DOX-PFP-PLGA nanodroplets. Then, DOX-PFP-PLGA could be condensation conjugated at the surface of bacteria *E. coli MG1655* (*EcM*) to fabricate ultrasound-responsive SonoBacteriaBot known as DOX-PFP-PLGA@*EcM*. SonoBacteriaBot will be transported to the tumor region *via* bacterial self-propulsion. Notably, it can be visualized by an echogenic signal from the nanodroplet-transition microbubbles (Alapan et al., 2018), which transition from DOX-PFP-PLGA, and the microbubbles will expand and collapse under continuous intensity ultrasound radiation, allowing for controlled drug release. Using SonoBacteriaBot’s ultrasound visualization and drug-controlled release, we can track the location of the bacteria-driven biohybrid microbots in real-time and achieve ultrasound-trigger drug release in the tumor site.

## Materials and methods

### Materials

Terminal carboxyl poly (lactic-glycolic acid) copolymer PLGA-COOH was purchased from Ruixi Biotechnology Co., Ltd. (Xian, China). Perfluorobutane (PFP), doxorubicin (DOX), and poly (vinyl alcohol) (PVA, MW = 25,000) were purchased from Aladdin Co., Ltd. (Shanghai, China). Chloroform (CHCl_3_) and isopropyl alcohol were purchased from Sangon Biotech Co., Ltd. (Shanghai, China). MES buffer was purchased from Solarbio (Beijing, China). 1-Ethyl-3-(3-(dimethylamino)propyl) carbodiimide (EDC) and n-hydroxysuccinimide (sulfo-NHS) were purchased from Sigma (United States). Fluorescein isothiocyanate (FITC), 1,1-dioctadecyl-3,3,3,3-tetramethylindotricarbocyanine iodide (DiR) were purchased from Beyotime Biotechnology Ltd., Co. (Shanghai, China). The pUC57/m-Cherry and *E. coli MG1655* (*EcM*) were purchased from Wuhan Miaoling Bioscience & Technology Co., Ltd. (Wuhan, China).

### Synthesis of PFP-PLGA/DOX-PFP-PLGA

All PFP-PLGA was prepared *via* the double-emulsion method (water/oil/water, W/O/W), and the steps are as follows. Initially, 40 mg carboxyl-modified PLGA was completely dissolved in 2 mL CHCl_3_ on ice. Following that, PFP was added to a final concentration of 10% (v/v in CHCl_3_), and the mixture was emulsified for 3 min using an ultrasonic disruptor (BIOBASE, China) to obtain the primary W/O emulsion. The power ratio of the ultrasonic disruptor is 100%. Subsequently, the primary W/O emulsion was then mixed with 4 mL of a PVA solution (w/v = 4%) and emulsified again to form the W/O/W double-emulsion. To solidify the particles, 10 mL of 2% isopropyl alcohol solution was added and stirred for 4 h, until CHCl_3_ was completely volatilized. The obtained particles were collected after repeated centrifugal rinsing with double steam water (10,000 rpm, 8 min), the supernatant was removed and precipitation was finally suspended in pre-cooling phosphate-buffered saline (PBS). PFP-PLGA encapsulating DOX was fabricated as described above, except that 2 mg DOX was similarly dissolved in CHCl_3_, initially. A common dye FITC label PFP-PLGA to prepare the FITC-PFP-PLGA was fabricated using the same method.

### Characterization of PFP-PLGA/DOX-PFP-PLGA

The morphology and structure of the PFP-PLGA and DOX-PFP-PLGA were characterized by transmission electron microscope (TEM, Hitachi H-7600, Japan) and the confocal laser scanning microscope (CLSM, LSM 880, Zeiss, United States), respectively. In addition, the UV absorption spectra of the PFP-PLGA, DOX-PFP-PLGA, and DOX were obtained using a UV-vis spectrophotometer (UV-3600, Shimadzu, Japan). The optical absorption properties of DOX (in ethyl alcohol) and calculation of the loading efficacy of DOX loaded in PFP-PLGA were employed *via* a UV-vis spectrophotometer. The encapsulation efficiency (EE) and loading capacity (LC) of DOX in the DOX-PFP-PLGA were calculated as previously described (Zhang et al., 2018). To confirm the physical stability of PFP-PLGA and DOX-PFP-PLGA, they were measured with DLS at 4°C for 6 days, respectively (Xi et al., 2022).

### Establishment and characterization of SonoBacteriaBot

*EcM* has been isolated from Luria broth (LB) agar plates and inoculated in freshly sterilized LB liquid medium for 4–8 h at 37°C and 220 rpm. *EcM* was collected by centrifugation (5,000 rpm, 4 min) when the optical density at 600 nm (OD600) was 0.5, and suspended in PBS for the fabrication of SonoBacteriaBot. The formation approach is to covalently couple carboxylated DOX-PFP-PLGA to *EcM* using carbodiimide chemistry as described previously (Taherkhani et al., 2014). The DOX-PFP-PLGA was suspended in 2 mL MES buffer (0.1 M, pH = 5.5), EDC, and sulfo-NHS were added respectively, corresponding to an EDC: sulfo-NHS: -COOH molar ratio of 30:30:1. After incubated at room temperature for 1 h to activate the carboxyl of PLGA, DOX-PFP-PLGA was centrifuged at 10,000 rpm for 5 min and washed with PBS to remove residual EDC and sulfo-NHS. The centrifuged precipitates were washed three times and then added into MG1655 solution and allowed to incubate for 2 h to obtain the SonoBacteriaBot (DOX-PFP-PLGA@*EcM*). All the above operations take place on the ice. After centrifugation (5,000 rpm, 5 min), SonoBacteriaBot was suspended with pre-cool PBS (pH = 7.4) and reserved at 4°C.

To evaluate the nanodroplets’ attachment efficacy to *EcM*, the connection between FITC-PFP-PLGA and mCherry-*EcM* was determined by a confocal laser microscope. Engineered *EcM* expressing the mCherry red fluorescent protein for indicating bacteria, and construction the FITC-PFP-PLGA showing green fluorescence for indicating nanoparticles. Flow cytometry was used to determine the bonding efficiency of FITC-PFP-PLGA to *EcM*, and the results were quantified. To detect the effect of DOX-PFP-PLGA on bacterial viability, the smear plate culture of *EcM* and DOX-PFP-PLGA@*EcM* containing the same amount of bacteria*,* and the number of bacterial colonies in the petri dish was counted.

### Animal model

All BALB/c mice (6–8 weeks, female) were purchased from the Hunan SJA Laboratory Animal Co., Ltd. (Hunan, China) and kept in a suitable temperature and humidity condition. All the experimental procedures were approved by the Medical Ethics Committee of the University of South China. The procedures involving animals were conducted with the ethical principles of the Experimental Animal Welfare Ethics Committee of the University of South China. Each mouse was respectively subcutaneously injected with 1 × 10^6^ 4T1 cells suspended in 100 L of PBS in the right flank to establish the 4T1 tumor mice models.

### Ultrasound imaging

A Sonovitro ultrasonic irradiator instrument (ShengXiang, China) was used for US treatment. Ultrasound scanning system (SonoKang, China) and High-resolution small animal ultrasound imaging system (S-Sharp, United States) for US imaging *in vitro* or *in vivo*, respectively. The ultrasound signals were recorded before and after the ultrasound treatment.

For *in vitro* US imaging, the effect of ultrasound intensity on ultrasound imaging of SonoBacteriaBot has been studied, DOX-PFP-PLGA@*EcM* (1 mL, 1.25 mg/mL) placed in a gel model, were exposed to US irradiation with different intensities (1–3 W/cm^2^, 1 min, 1 MHz), and then captured US imaging (MI = 0.7, 7.5 MHz). To evaluate the effect of ultrasound treatment time on the imaging effect of SonoBacteriaBot *in vitro*. DOX-PFP-PLGA@*EcM* was subjected to ultrasonic irradiation of different durations (0–10 min, 1 W/cm^2^, 1 MHz), and captured US imaging. Then, Imaging of DOX-PFP-PLGA@*EcM* after phase transformation by ultrasound treatment under an ultrasound intensity of 1 W/cm^2^ for 2 min, was observed continuously (0–10 min). Ultrasound images were acquired at different times to evaluate the duration of the imaging signal.

For *in vivo* US imaging, mice were intravenously injected with DOX-PFP-PLGA @*EcM* in PBS (100 μL, 2.5 mg/mL). At 1 h after injection, the liver was irradiated using US (1 W/cm^2^, 10 min, 1 MHz). Subsequently, US imaging (40 MHz) of the liver imaging was performed. The corresponding echo intensity values of the ROI were quantitatively analyzed using ImageJ software.

### Ultrasound triggered drug release

The 24 well plates were filled with a certain amount of DOX-PFP-PLGA@*EcM* and then exposed to US radiation at various intensities of 1, 2, 3 W/cm^2^, and at various periods of 1, 3, 5, 10 min for experiments on *in vitro* drug release. Supernatants from centrifuged samples were used to determine the amount of drug released after samples were taken at regular intervals. The same approach was used to conduct comparative research without the US to assess the consequences of the US-triggered release. In addition, to detect the US irradiation of DOX-PFP-PLGA@EcM on bacterial viability, the smear plate culture of US irradiated and non-irradiated DOX-PFP-PLGA@EcM with the same amount of bacteria, and the number of bacterial colonies in the petri dish was counted.

### Targeting ability and biodistribution of SonoBacteriaBot

To evaluate the targeting ability and biodistribution *in vivo* of SonoBacteriaBot, the 4T1 tumor-bearing BALB/c mice were injected with DOX-PFP-PLGA and DOX-PFP-PLGA@*EcM* (100 μL, 2.5 mg/mL) through the tail vein, respectively. After injection, the mice were anesthetized and imaged with Maestro Automated *in vivo* Imaging at different points (0, 1, 6, 12, 18, 24, and 48 h post-injection). After that, the mice were sacrificed to obtain the main organs and tumors for *ex vivo* tissue fluorescence imaging. Besides, the tissues were ground and cultured on agar plates under a suitable environment (37°C), and then calculate the number of colonies was for bacterial distribution evaluation *ex vivo*.

### Biocompatibility evaluation of SonoBacteriaBot

*In vivo* studies were carried out on BALB/c mice, who were then dissected after receiving various treatments. To investigate the systemic toxicity, a portion of the key organs (heart, liver, spleen, lung, and kidney) were stained with H&E. Additionally, following DOX-PFP-PLGA@*EcM* with US therapy, serum aspartate aminotransferase (AST)/alanine aminotransferase (ALT) measurements were used to assess the liver function, and serum urea nitrogen (UREA)/creatinine (CREA) measurements were used to assess the kidney function.

### Statistical analysis

All data were analyzed by one-way analysis of variance (ANOVA), and Student’s *t*-tests with GraphPad Prism 7. *p < 0.05 was considered significant for the differences.

## Result

### Synthesis and characterization of PFP-PLGA/DOX-PFP-PLGA

**FIGURE 2:**
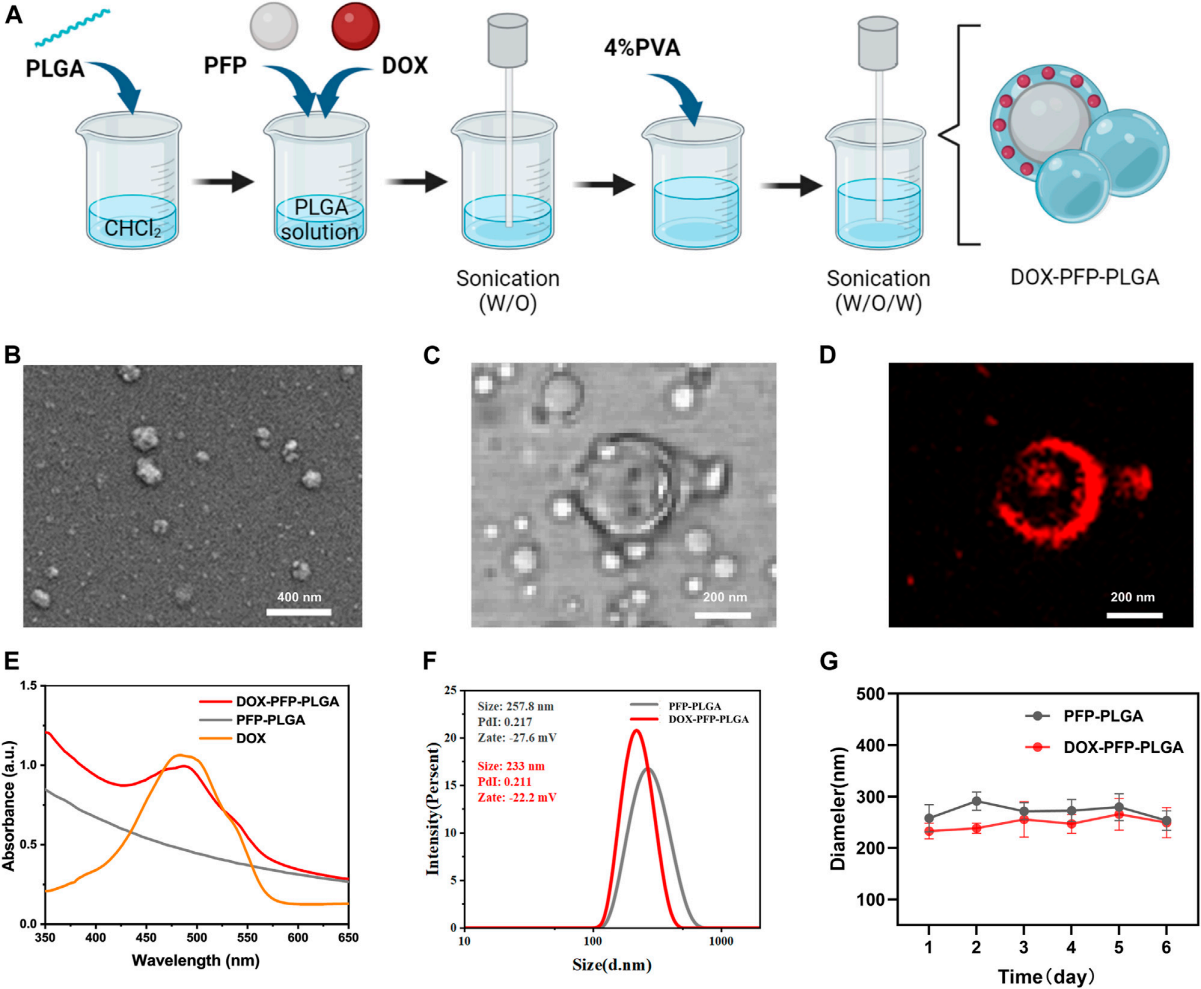
Synthesis and characterization of PFP-PLGA and DOX- PFP-PLGA. **(A)** The schematic diagram of the synthesis of DOX-PFP-PLGA (created with Biorender.com). **(B)** TEM image showed the morphology of PFP-PLGA. **(C,D)** CLSM image of DOX-PFP-PLGA. **(E)** UV-vis absorption spectra of DOX-PFP-PLGA, PFP-PLGA, and DOX. **(F)** The size distribution and zeta potential of PFP-PLGA and DOX- PFP-PLGA. **(G)** The size distribution with prolonged time duration of PFP-PLGA and DOX- PFP-PLGA.

The double-emulsion method was used to synthesize ultrasound-responsive PFP-PLGA and DOX-PFP-PLGA in our study (Figure 2A). The PFP-PLGA exhibited a nanosized sphere (Figure 2B) and displayed a mean size of 257.8 ± 2.80 nm, an average zeta potential of −27.6 ± 1.2 mV (Figure 2F). PFP-PLGA were allowed to encapsulate, carry and release a drug upon ultrasound-mediated heat effect at a specific site with appropriate design. The DOX-PFP-PLGA was prepared with a PLGA shell and contained the compartment carrying PFP and drugs such as DOX. Since DOX has red fluorescence, we can observe the red fluorescence of DOX-loaded PFP-PLGA under CLSM (Figures 2C, D). According to DLS measurements, the final DOX-PFP-PLGA size was 233 ± 3.4 nm, with an average zeta potential of −22.2 ± 1.2 mV (Figure 2F).

The UV absorption spectra showed DOX has a characteristic peak at 480 nm. To further prove the loading of DOX, the UV-vis spectrum of DOX-PFP-PLGA was compared with that of PFP-PLGA and pristine DOX. The result showed that DOX was successfully encapsulated in the nanodroplets (Figure 2E). Additionally, according to the standard curve of DOX, EE and LC of DOX in DOX-PFP-PLGA were calculated to be (47.31% ± 3.54%) and (4.73% ± 0.35%), respectively. The result showed that PFP-PLGA could carry water-soluble drugs. There is no significant difference between the particle sizes of PFP-PLGA and DOX-PFP-PLGA in PBS at 4°C for 6 days, which showed PFP-PLGA and DOX-PFP-PLGA possessed satisfactory structural stability (Figure 2G).

### Characterization of SonoBacteriaBot

**FIGURE 3:**
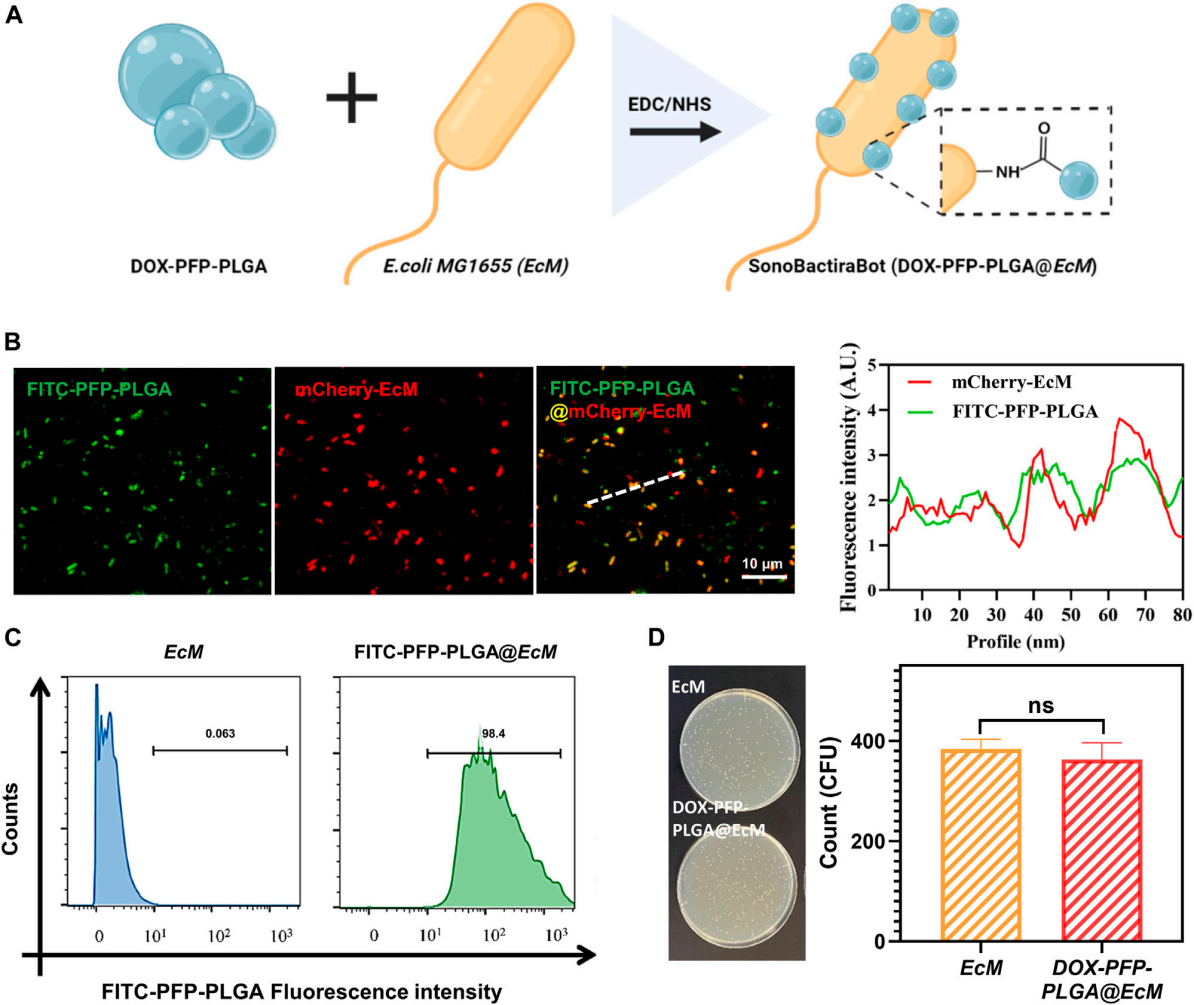
Construction and characterization of SonoBacteriaBot. **(A)** The schematic diagram of SonoBacteriaBot, showed DOX-PFP-PLGA attached to *EcM* (created with Biorender.com). **(B)** Confocal microscopy images and intensity of the underlined FITC-PFP-PLGA, mCherry-*EcM*, and FITC-PFP-PLGA @mCherry-*EcM*. **(C)** Flow cytometry histograms obtained from FITC-PFP-PLGA nanodroplets attended and non-attended *EcM.* **(D)** Live/Dead bacteria of DOX-PFP-PLGA nanodroplets attended and non-attended *EcM*.

Herein, we prepared a biohybrid microbot consisting of DOX-PFP-PLGA and *EcM* acting as an efficient vector to deliver DOX-PFP-PLGA (Figure 3A). Next, mCherry expressing *EcM* with red fluorescence was used to co-locate with FITC-PFP-PLGA (Green fluorescence), which led to the merged orange fluorescence and indicated the successful loading of PFP-PLGA on *EcM*. Each fluorescence intensity of green and red indicated a positive correlation between them (Figure 3B). Then, flow cytometry was carried out to further determine the attached efficiency of DOX-PFP-PLGA to the *EcM* surface. The fluorescence intensity in distribution histograms was presented by FlowJo software (Figure 3C). The result showed that DOX-PFP-PLGA were successfully connected to the surface of *EcM.* According to the reported method (Xiao et al., 2022), we next validated the effect of DOX-PFP-PLGA coating on bacterial activity. By counting the bacterial colonization in each plate, it was found that no statistical difference in the activity of bacteria attaches or non-attach nanodroplets (Figure 3D). The results indicate that the binding of DOX-PFP-PLGA onto *EcM* did not affect bacterial growth.

### Ultrasound imaging of SonoBacteriaBot

**FIGURE 4:**
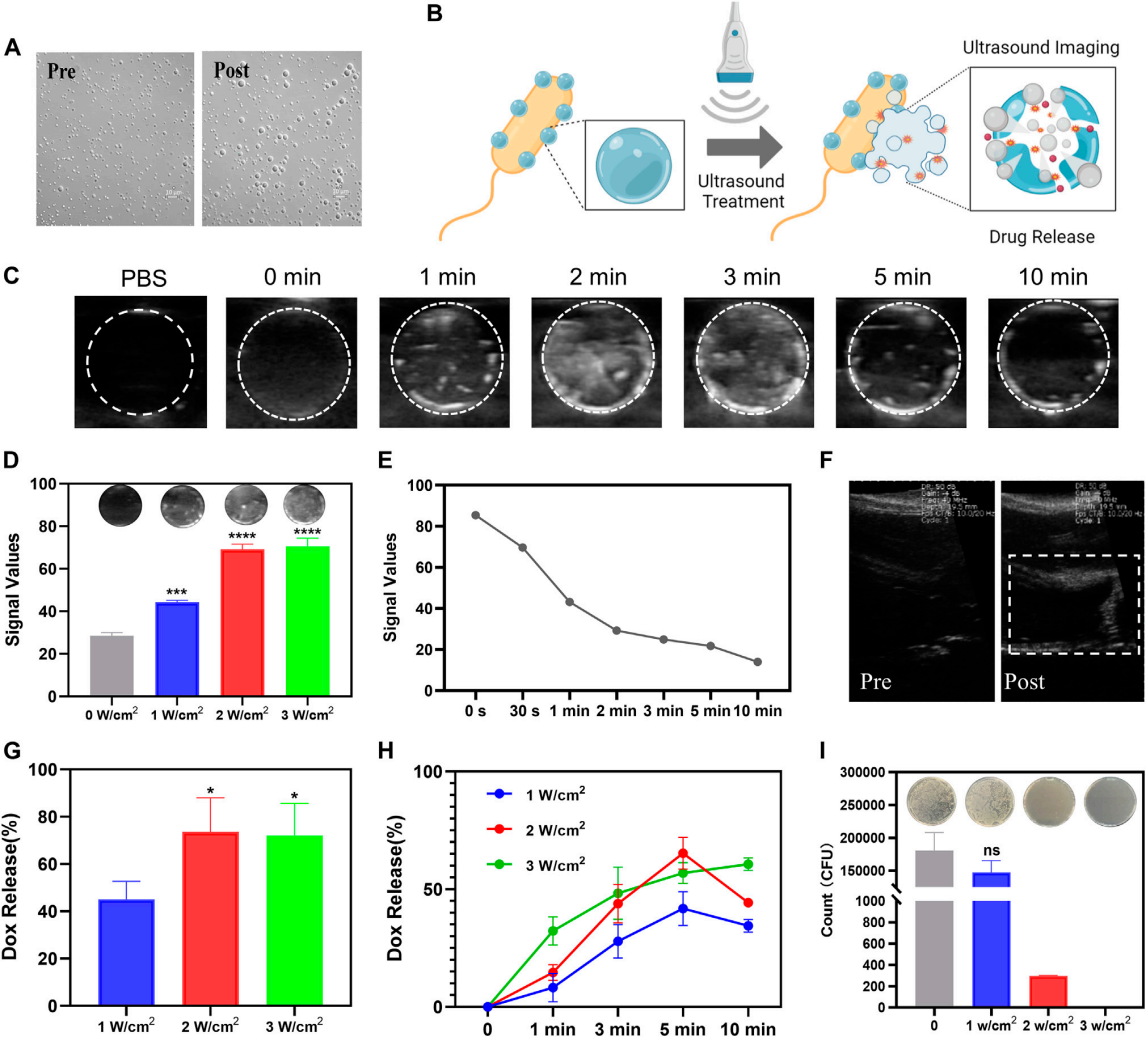
The controlled release of drugs under the guidance of ultrasound imaging of SonoBacteriaBot. **(A)** The transition from nanodroplets to microbubbles of PFP-PLGA. **(B)** The schematic diagram of ultrasound images and drug release of SonoBacteriaBot (created with Biorender.com). Ultrasound images of DOX-PFP-PLGA@*EcM* before and after US irradiation. **(C)** US images of DOX-PFP-PLGA@*EcM* irradiated using sonovitro irradiation (1 W/cm^2^, 1 MHz) for different durations (*n* = 3). **(D)** US images and echo intensity values of DOX-PFP-PLGA@*EcM* irradiated using sonovitro irradiation for different ultrasonic intensities (*n* = 3). **(E)** Duration of echo intensity values. **(F)** *In vivo* US images of mouse liver vascular. Ultrasound trigger drug release of DOX-PFP-PLGA@*EcM.* **(G)** Drug release profile of DOX from DOX-PFP-PLGA@*EcM* with different ultrasonic intensity (*n* = 3) for 3 min. **(H)** Drug release profile of Doxorubicin from DOX-PFP-PLGA@*EcM* with different ultrasonic intensities for different durations (*n* = 3). **(I)** Live/Dead bacteria of US irradiated and non-irradiated DOX-PFP-PLGA@*EcM*.

Based on the ability of PFP to induce acoustic phase change, we first investigated ultrasound-induced NPs phase change to evaluate the ultrasound imaging capability of PFP-PLGA. The imaging ability of PFP-PLGA nanoparticles before and after irradiation was observed under an optical microscope. After irradiation, the size of PFP-PLGA increases significantly (Figure 4A). Because of the low boiling point of PFP, DOX-PFP-PLGA vaporizes after being heated to physiological temperature by ultrasonic irradiation, and nanodroplets enlarged and fused into microbubbles. *In vitro* ultrasonic imaging simulation of DOX-PFP-PLGA@*EcM*, under ultrasonic irradiation, the bacteria carrying nanoparticles undergo liquid-gas phase transformation, forming large microbubbles. B-mode ultrasound was used for imaging to observe the imaging ability of bacterial nanomaterials. The results of the experiment showed the imaging effect of DOX-PFP-PLGA@*EcM* is related to ultrasound intensity and irradiation time. As shown in Figure 4D, ultrasound grayscale changes were observed after US irradiation of different ultrasound intensities. The ultrasonic echo intensity from the DOX-PFP-PLGA@*EcM* is gradually increased with the increase of ultrasound intensity. Within 0–10 min of US irradiation, the echo intensity from the DOX-PFPPLGA@*EcM*. Within 0–10 min of US irradiation, the echo intensity from the DOX-PFP-PLGA@*EcM* gradually increased and peaked at 2 min and then significantly decreased after 3 min (Figure 4C). After ultrasonic treatment, the signal intensity was continuously monitored. As the time increased, the signal values gradually decreased. This result indicated the imaging signal maintenance time of the bacterial complex (Figure 4E). *In vivo* ultrasound imaging was attempted, and we discovered that post-injection and ultrasound treatment allowed for experimental liver vascular imaging (Figure 4F).

### Ultrasound trigger drug release of SonoBacteriaBot

The exogenous ultrasound stimulus not only stimulated imaging but also triggered drug release (Figure 4B) (Amin et al., 2021). The DOX released from DOX-PFP-PLGA@*EcM* relied on ultrasound stimuli. DOX-PFP-PLGA@*EcM* exhibited rapid release of DOX, after the ultrasound due to the fast structural collapse. Notably, with the increase in the intensity and time of ultrasonic treatment, the DOX release efficiency showed a rising trend (Figures 4G, H), which is consistent with the imaging results. Besides, the results showed that the effect of drug release was rather reduced when the intensity was greater than 2 W/cm^2^ or the treatment time exceeded 5 min. This may be attributed to a certain degree of sample depletion during ultrasonic treatment. The experiments show evidence that DOX-PFP-PLGA@EcM exposure to the US can trigger the release of DOX. To determine the effect of ultrasound on DOX-PFP-PLGA@*EcM’s* activity, we conducted experiments and found that the bacterial activity could be roughly retained after 1 W/cm^2^ treatment, while it was significantly damaged after 2 and 3 W/cm^2^ treatment (Figure 4I).

### *In vivo* distribution of SonoBacteriaBot

**FIGURE 5:**
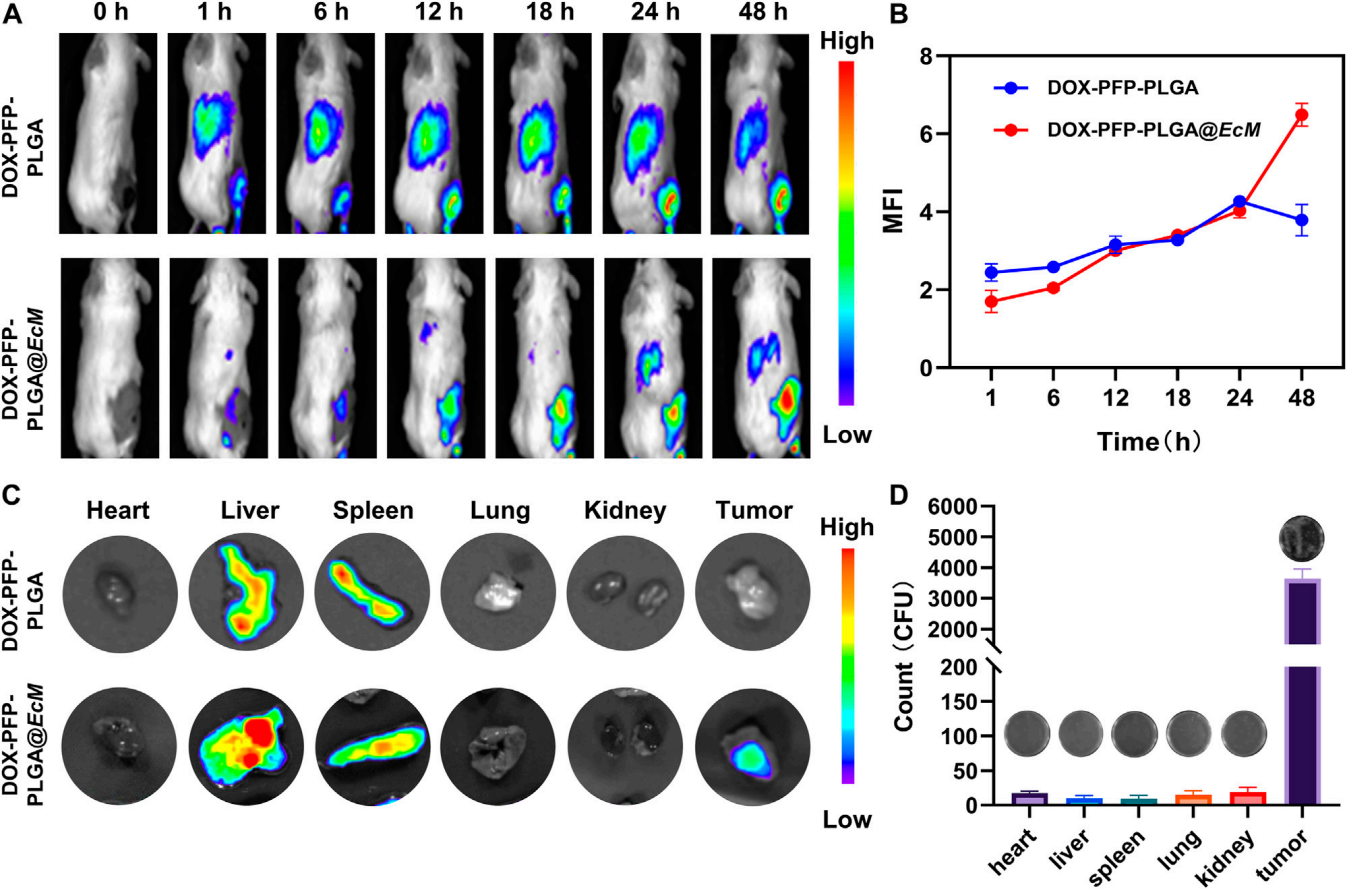
*In vivo* distribution of DOX-PFP-PLGA@*EcM*. **(A)** *In vivo* fluorescence imaging of 4T1 tumor-bearing mice at different injection times of DOX-PFP-PLGA and DOX-PFP-PLGA@*EcM* and **(B)** The ratio of the real-time signal intensity and the before injection intensity is used as the measuring FL values in different time points. **(C)** The bio-distribution of DOX-PFP-PLGA or DOX-PFP-PLGA@EcM in major organs and tissue excised from mice at 48 h post-injection. **(D)** Homogenates of tumors and organs were cultured on solid agar at 37°C.

To determine the capability of tumor targeting of bacteria, the DiR labeled DOX-PFP-PLGA and DOX-PFP-PLGA@*EcM* were used *in vivo*. DOX-PFP-PLGA and DOX-PFP-PLGA@*EcM* were injected intravenously into 4T1 tumor-bearing mice, respectively. After 12 h post-injection, DiR signals were detected within the tumor of the DOX-PFP-PLGA@*EcM* group (Figure 5A). Then, the fluorescence intensity of the tumor site in the DOX-PFP-PLGA@*EcM* group was 1.9 times higher than that in the DOX-PFP-PLGA group at 48 h after injection (Figure 5B). 48 h after the injection, the primary organs and tumors were excised for fluorescent imaging *in vitro*. Due to the phagocytosis of the reticuloendothelial system, significant fluorescence signals in both groups highlighted the liver and spleen. Notably, the tumor’s fluorescence intensity in the DOX-PFP-PLGA@*EcM* group was noticeably higher than that in the DOX-PFP-PLGA group (Figure 5C). Furthermore, the *EcM* in each major organ and tumor tissue was quantitated by counting bacterial colonies in the DOX-PFP-PLGA@*EcM* group. We found that the number of bacteria in tumors was significantly higher than in other organs (Figure 5D). The results proved that DOX-PFP-PLGA@*EcM* allowed for effective accumulation in tumor tissue and enhance the drug delivery effect.

### Biosafety evaluation

**FIGURE 6:**
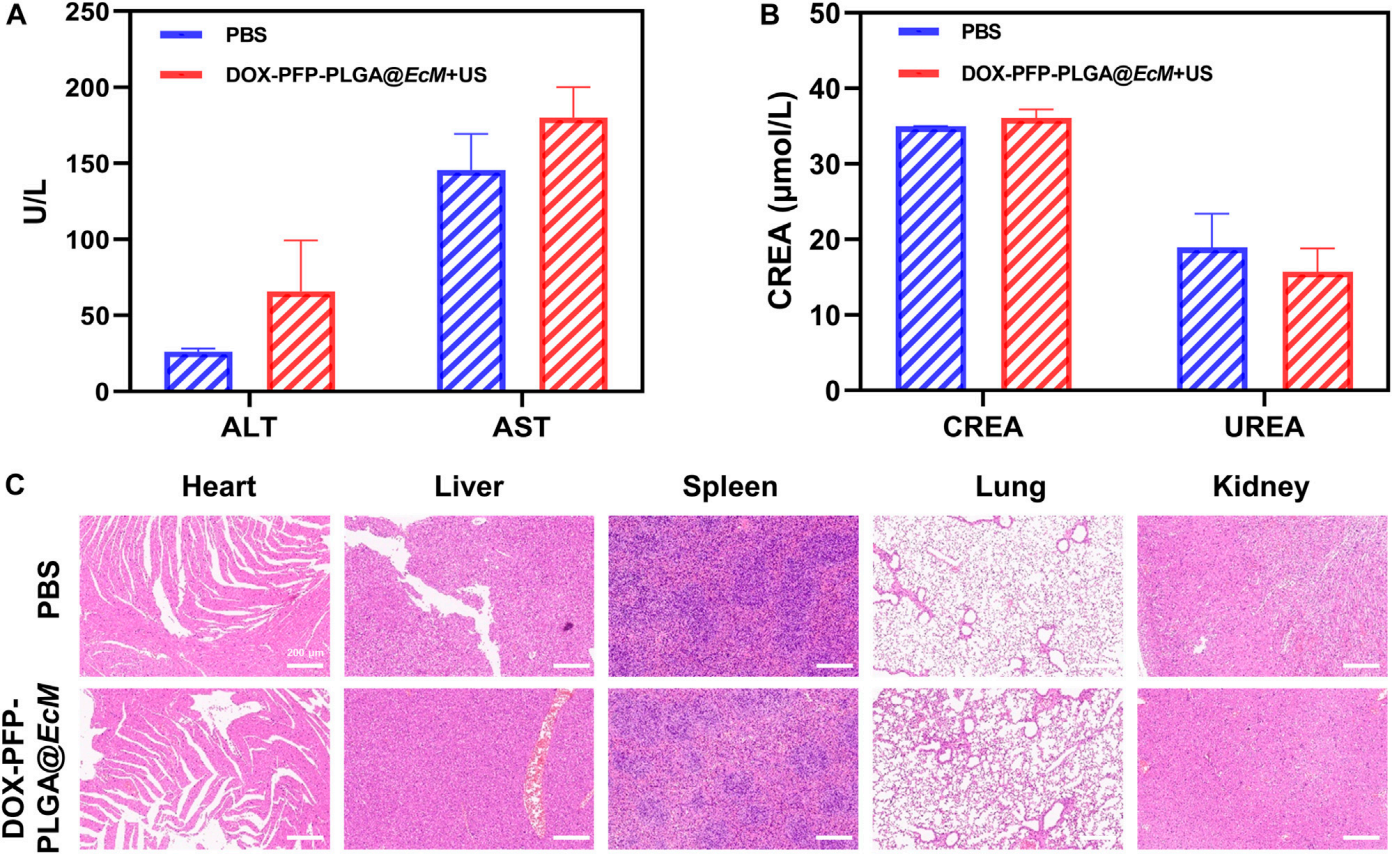
Biosafety Evaluation. **(A)** Serum levels of ALT and AST (liver function) (*n* = 3). **(B)** Serum levels of creatinine (CREA) and urea (UREA) (renal function, *n* = 3). **(C)** Histological analysis *in vivo*. H&E staining of heart, liver, spleen, lung, and kidney from the 4T1 tumor-bearing mice at 48 h after injection.

Biosafety is a common concern, and this cancer treatment model’s safety was finally evaluated in mice. The liver and kidney function test results showed that all biochemical indicators fluctuated within the normal range and had no significant difference compared with the untreated group (Figures 6A, B). Furthermore, H&E staining of the heart, liver, spleen, lung, and kidney showed no histopathological lesions (Figure 6C). As a result, these findings demonstrate the therapeutic biosafety of DOX-PFP-PLGA@*EcM in vivo*.

## Discussion

In this work, we have designed an ultrasound-responsive SonoBacteriaBot for ultrasound-regulated drug release and imaging guidance. SonoBacteriaBot could selectively accumulate at the tumor site upon systemic treatment, making it possible to locally trigger acoustic phase change and drug release under US irradiation.

Our research confirms that SonoBacteriaBot has the feature of imaging under the action of ultrasound, and the imaging signal is positively correlated with ultrasound sound intensity and processing time, to a certain extent. This is mainly due to fluorocarbon NPs in them, which can strongly absorb ultrasonic energy and generate heat, resulting in liquid-gas phase transition and bubble formation, which are beneficial to perform for ultrasound imaging (Wang et al., 2012; Jiang et al., 2022). However, in the next study, we found the imaging signal gradually declined after 5 min with the extension of ultrasound treatment time. It may be due to a certain amount of DOX-PFP-PLGA@*EcM* undergoing phase change and loss, resulting in poor subsequent imaging after 5 min. This is similar to the previous result that Ran et al. (Yao et al., 2021) also studied the fluorocarbon NPs irradiated with LIFU for 0–6 min, and the echo intensity of B-mode gradually increased and reached the peak value during 4 min, and decreased significantly after 5 min. Although DOX-PFP-PLGA@*EcM* has an obvious imaging effect *in vitro*, *in vivo* imaging is not satisfactory. The imaging function of DOX-PFP-PLGA@*EcM* is derived from nanodroplets. However, the limitation of nanodroplets carried by bacteria and their circulation metabolic effects *in vivo* leads to suboptimal tumor imaging, which can now only be achieved in the vascular-rich region of the liver. Therefore, optimizing the connection between bacteria and nanodroplets is necessary to improve the loading of nanoparticles and enhance the imaging effect.

Efficient controlled cargo release methods that are an effective aid in cancer treatment. Previously, most studies utilized the pH sensitivity of nanoparticles to release the drug in an acid tumor microenvironment. However, not all the solid tumors had a consistent and stable pH environment (Park et al., 2017; Xie et al., 2017; Alapan et al., 2018). This controlled release effect is limited because the pH sensitivity will decrease as the copolymer dosage increases (Salve et al., 2021). A release profile under external stimulation, such as ultrasound, will provide a promising solution to the above-mentioned delivery problem. Our study confirms that DOX-PFP-PLGA@*EcM* can be used not only for ultrasound imaging but also for ultrasound-mediated controlled drug release. This is primarily because the vaporization of PFP by US mechanical, thermal, or mixed forces results in a significant volume of gas being created. In addition to rupturing the PLGA coating, this gas pressure also caused the medication to be released mechanically (Sirsi and Borden, 2014). Notably, the results suggest that DOX-PFP-PLGA@*EcM* showed a relationship between the ultrasound imaging signal the drug release of DOX, which provided a possibility for US-guided triggered drug release. In addition, the cavitation reaction of vaporized PFP-PLGA, which is capable of propelling therapeutic drugs deeper into surrounding tissues *via* radiative forces and microstreaming, is expected to enhance this localized release.

It is important to note that *in vivo* toxicity remains a concern for the clinical translation of bacteria-mediated therapy. Our biosafety research showed the SonoBacteriaBot’s ultrasound-triggered delivery mode is reliable and safe, with little impact on normal tissue.

## Conclusion

Herein, we designed and constructed the SonoBacteriaBot (DOX-PFP-PLGA@*EcM*), an ultrasound-responsive device made up of a living microbot and an echogenic drug-carrying vehicle. Based on the tumor-targeting ability of *EcM*, DOX-PFP-PLGA was delivered to the tumor to enhance the nanoparticle delivery efficiency. Based on the acoustic phase change capability of PFP, DOX-PFP-PLGA@*EcM* can trigger the acoustic phase change of nanoparticles under ultrasound treatment to achieve ultrasound imaging capability and further controlled drug release. Ultrasound-responsive SonoBacteriaBot provides great advantages in controlled drug release and real-time monitoring of microbots. It has great potential for clinical chemotherapy drug delivery.

## Data Availability

The raw data supporting the conclusion of this article will be made available by the authors, without undue reservation.
